# Supplementary material for: DUOX2-Induced Oxidative Stress Inhibits Intestinal Angiogenesis through MMP3 in a Low-Birth-Weight Piglet Model
Source: Antioxidants (Basel). 2023 Sep 25;12(10):1800. doi: 10.3390/antiox12101800 (PMC10603984; doi:10.3390/antiox12101800)
Supplement: Supplementary file 1 [file antioxidants-12-01800-s001.zip › antioxidants-2500836-supplementary.pdf]

Supplementary Table S1. Primers used for real-time PCR

Genes	Primers	Sequences (5′ to 3′)
ATF4	Forward	AACATGGCCGAGATGAGCTTCC
	Reverse	TCTCCACCATCCAGTCTGTCCC
ATF6	Forward	CTCAGCTCATGGCTGTCCAA
	Reverse	AATGTGTCTCCCCTTCTGCG
bFGF	Forward	TCAAAGGAGTGTGTGCGAAC
	Reverse	CAGGGCCACATACCAACTG
CD31	Forward	AACCTCGCCCATTTCCTACC
	Reverse	CTGTTTTCCACTAAATCAGGGTC
CHOP	Forward	TTAAGTGTGACAAGGAGAAGAAC
	Reverse	CAAGGAAGGCAGAATAGAAGC
CytB	Forward	ATGAAACATTGGAGTAGTCCTACTATTTACC
	Reverse	CTACGAGGTCTGTTCCGATATAAGG
Claudin 1	Forward	CTAGTGATGAGGCAGATGAA
	Reverse	AGATAGGTCCGAAGCAGAT
GRP78	Forward	AGTCCCGCAGATTGAAGTCA
	Reverse	TCTTCAGGTGTCAGGCGATT
MMP2	Forward	ACTCCCACTTTGACGACGAT
	Reverse	CGTACTTGCCATCCTTGTCG
MMP9	Forward	GGTGGACTATGTGGGCTACG
	Reverse	AGTGCTGAAGCAGGACGAG
SLC1A5	Forward	GATTGTGGAGATGGAGGATGTGG
	Reverse	TGCGAGTGAAGAGGAAGTAGATGA
SLC7A2	Forward	TGCCCATACTTCCCGTCC
	Reverse	GGTCCAGGTTACCGTCAG
SLC7A5	Forward	TTTGTTATGCGGAACTGG
	Reverse	AAAGGTGATGGCAATGAC
SLC7A7	Forward	TTTGTTATGCGGAACTGG
	Reverse	AAAGGTGATGGCAATGAC
SLC7A9	Forward	CACAACAACTGCGAGAAGGA
	Reverse	CCGTTGATAAGCGTCAGGAT
SLC36A1	Forward	TGTGGACTTCTTCCTGATTGTC
	Reverse	CGTTGTTGTGGCAGTTGTTGGT
SLC38A2	Forward	TACTTGGTTCTGCTGGTGTCC
	Reverse	GTTGTGGGCTGTGTAAAGGTG
Occludin	Forward	GAGTGATTCGGATTCTGTCT
	Reverse	TAGCCATAACCATAGCCATAG
PDGF-C	Forward	GGAGTACAAGATCCCCAGCA
	Reverse	TCCAGCCCAAATCTCTCATC
TGF-1β	Forward	CAAGGTCCTGGCTCTGTACA
	Reverse	CAGGAACGCACGATCATGTT
VEGF-A	Forward	CCTCGGAGCGGAGAAAGCAT

	Reverse	TGTCACATCTGCAAGTACGTTCG
18S rRNA	Forward	ATTCCGATAACGAACGAGACT
	Reverse	GGACATCTAAGGGCATCACAG
XBP-1	Forward	GCCTCCCCTTCTTCATCACT
	Reverse	TTTCTCTGAGGGGCTGGAAG
Keap1	Forward	CGTGGAGACAGAAACGTGGA
	Reverse	CAATCTGCTTCCGACAGGGT
GLUT1	Forward	CCTTCAGCCAGCAGTGATG
	Reverse	AGCGTGGGATGTGGGTAAAG
GLUT3	Forward	GCCTTGACCTTTCCCATAGACA
	Reverse	CTACTTCCACCCAGAGCAAAGT
GLUT4	Forward	TCTCCAACTGGACCTCGAATTT
	Reverse	CCGCACAGTTGCTCCACATA
VEGFR2	Forward	GATGCTCGCCTCCCTTTGA
	Reverse	AGTTCCTTCTTTCAGTCGCCTACA

Table S2. Differentially expressed genes in jejunum between CON and LBW groups

	LBW group					CON group						
Gene_name	JL1_fpkm	JL2_fpkm	JL3_fpkm	JL4_fpkm	JL5_fpkm	JN1_fpkm	JN2_fpkm	JN3_fpkm	JN4_fpkm	JN5_fpkm	JNvsJL_log2 Fold Change	JNvsJL *P*-value
NOX1	0.013	0.000	0.000	0.104	0.061	0.000	0.410	0.077	0.000	2.047	3.823	0.058
NOX3	0.000	0.000	0.000	0.000	0.000	0.000	0.000	0.000	0.000	0.000	-	-
NOX4	0.335	0.567	0.273	1.021	0.616	1.278	0.495	0.638	0.938	1.003	0.629	0.113
NOX5	0.135	0.584	0.261	0.041	0.217	0.230	0.520	0.314	0.150	0.521	0.492	0.397
DUOX1	0.000	0.000	0.000	0.000	0.000	0.000	0.000	0.000	0.000	0.000	-	-
DUOX2	18.062	0.600	8.343	83.877	16.581	1.733	2.877	1.290	2.722	8.557	-2.891	0.003
DUOXA2	3.532	0.137	1.390	15.368	2.673	0.432	0.272	0.092	0.491	0.693	-3.544	0.000
FGF1	2.398	1.422	1.947	2.224	1.769	0.973	0.572	0.113	1.168	1.167	-1.289	0.005
VEGFA	23.559	33.182	25.221	22.659	20.408	34.903	31.185	42.300	68.509	41.745	0.807	0.001
FGFBP1	19.668	11.178	8.812	24.590	22.534	0.471	0.473	0.112	1.815	1.119	-4.440	0.000
FGFR3	15.056	20.959	20.447	16.218	25.285	30.852	38.988	47.646	44.600	41.721	1.057	0.000
IGF1	0.454	0.292	0.232	0.443	0.297	1.777	0.948	0.435	0.270	0.531	1.204	0.012
IGF2R	12.500	15.690	11.754	14.451	13.525	27.421	24.313	33.605	18.894	19.599	0.866	0.000
ETFDH	11.303	14.540	12.388	12.163	15.488	14.661	20.250	17.751	19.042	10.799	0.325	0.129
GCH1	3.386	7.611	5.473	4.293	7.996	8.203	16.462	18.822	13.158	11.900	1.253	0.000
NQO1	41.871	13.491	8.244	80.811	12.666	7.040	7.410	8.485	7.633	5.128	-2.138	0.000
PNPT1	4.948	16.942	7.523	8.072	14.326	4.127	4.351	2.930	5.011	4.343	-1.319	0.000
SOD3	26.777	28.782	20.894	8.169	20.827	43.656	32.178	57.557	33.648	23.738	0.856	0.014
CAT	18.941	40.920	20.919	24.513	32.843	74.450	60.607	77.189	38.785	34.881	1.050	0.001
OCEL1	28.175	22.316	28.752	13.933	15.823	32.909	35.755	62.240	46.342	31.776	0.940	0.001
CLDN15	0.720	0.899	1.060	0.647	0.556	1.774	1.589	1.230	1.017	2.008	0.977	0.000
CD58	4.847	7.218	4.370	6.590	6.733	10.404	11.041	9.956	8.119	8.038	0.676	0.001
KRT8	1426.726	588.146	732.995	633.978	920.243	291.771	423.863	417.701	624.388	561.813	-0.891	0.003
CLDN3	630.085	680.760	864.789	361.999	675.145	559.250	793.201	1348.493	1444.648	1293.074	0.759	0.016
PRDX5	15.691	14.971	12.903	8.873	17.216	23.625	30.155	39.958	30.796	22.764	1.080	0.000
PRDX6	86.702	58.153	85.012	145.123	76.970	47.212	50.462	60.363	59.425	36.699	-0.831	0.002
PRDX6	86.702	58.153	85.012	145.123	76.970	47.212	50.462	60.363	59.425	36.699	-0.831	0.002

PRDX1	344.044	433.557	365.394	293.451	432.768	238.192	279.550	265.065	335.725	254.755	-0.445	0.013
